# Critical assessment of the latest classification of jaw cysts proposed by the World Health Organization (2017)

**DOI:** 10.4317/jced.58764

**Published:** 2021-11-01

**Authors:** Kevin Barrios-Garay, Luisa-Fernanda Agudelo-Sánchez, José-Manuel Aguirre-Urizar, Cosme Gay-Escoda

**Affiliations:** 1 Dentistry student. Faculty of Medicine and Health Sciences, University of Barcelona, Barcelona, Spain; 2 MD, DDS, PhD. Chairman and Professor of Oral Medicine, Department of Stomatology II, Faculty of Medicine and Nursery, University of the Basque Country/EHU, Leioa, Spain; 3 MD, DDS, MS, PhD, EBOS, OMFS. Chairman and Professor of Oral and Maxillofacial Surgery, Faculty of Medicine and Health Sciences, University of Barcelona. Director of the Master Degree Program in Oral Surgery and Implantology (EHFRE International University/FUCSO). Coordinator/Researcher at the IDIBELL Institute. Head of the Department of Oral Surgery, Implantology and Maxillofacial Surgery, Teknon Medical Center, Barcelona, Spain

## Abstract

**Background:**

The fourth edition of the World Health Organization Classification of Head and Neck Tumors was published in January 2017, and includes a classification of odontogenic tumors and odontogenic cysts. The present review assesses the changes made in this new classification in relation to odontogenic and non-odontogenic jaw cysts.

**Material and Methods:**

An electronic search was conducted in the Cochrane Library, PubMed-MEDLINE and Scopus databases using the search terms: “odontogenic cyst” “WHO classification” “update”. Studies written in English and published between January 2005 and April 2020 with a high level of scientific evidence were included, while studies not published in English, epidemiological studies, and studies with a low level of scientific evidence were excluded.

**Results:**

The initial search identified 311 articles, and after the deletion of duplicates, 7 studies were selected for full-text assessment. After excluding two studies that failed to provide relevant information and had a low level of scientific evidence, 5 articles were finally included and stratified according to their level of scientific evidence based on the SORT (Strength of Recommendation Taxonomy) criteria.

**Conclusions:**

The incorporation of odontogenic and non-odontogenic cysts to the head and neck tumors classification underscores the recognition of the WHO of these important disorders of the jaws. Based on the current evidence, there is controversy as to whether odontogenic keratocysts should be regarded as cystic lesions or as neoplasms, though there is no such controversy in relation to calcifying odontogenic cysts. On the other hand, orthokeratinized odontogenic cysts have been included in the classification as a single entity differentiated from odontogenic keratocysts, while residual cysts have been removed from the classification.

** Key words:**Odontogenic cyst, WHO classification, pseudocyst.

## Introduction

The first internationally accepted classification proposed by the World Health Organization (WHO) in reference to odontogenic tumors and cystic lesions of the jaws was published in 1971 (1). This classification included odontogenic lesions, tumors and cysts, together with other jaw bone-related lesions that must be differentiated from odontogenic lesions.

In 1992, this classification of odontogenic lesions was revised (2), and in 2005 (3) cysts were removed from the WHO Classification. Pathology and Genetics of Head and Neck Tumors - probably because this was a classification focused on tumors and did not consider odontogenic cysts as such. Nevertheless, based on the scientific evidence at that time, the decision was made to reclassify some odontogenic cyst presentations as tumor lesions. Accordingly, odontogenic keratocysts and calcifying odontogenic cysts were respectively classified as keratocystic odontogenic tumors and calcifying cystic odontogenic tumors (3).

This classification considered that odontogenic cysts should not be included. Nevertheless, it recognized that bone cystic lesions were relevant for the differential diagnosis of jaw lesions (4).

The ninth volume of the fourth edition of the World Health Organization Classification of Head and Neck Tumors was published in January 2017 under the guidance of different worldwide experts with experience in the study of these disorders (5).

In this latest classification of 2017 (5), the list of tumor entities proposed in 2005 (3) has been simplified, and odontogenic and non-odontogenic cysts of the jaws have been reintroduced, with a significant update from the classification of 1992 (2).

Odontogenic cysts of inflammatory origin are differentiated from odontogenic and non-odontogenic developmental cysts (5). Outside this section, and within the group of “giant cell lesions and bone cysts”, simple bone cysts and aneurysmal bone cysts - two pseudocystic entities - are cited and must be taken into account in the differential diagnosis of cystic disease of the jaws (5).

The primary objective of the present study was to analyze the main changes that have taken place in the new WHO classification of 2017 (5) in relation to cystic lesions of the jaws.

## Material and Methods

An electronic search was conducted between February and April 2020 in the Cochrane Library, Scopus and PubMed-MEDLINE databases using the search terms: “odontogenic cyst”, “WHO classification” and “update”. Search terms were combined using the Boolean operator “AND”, with the aim of obtaining different articles that included two or more of the terms used for the search.

We included all studies published between January 2005 and April 2020 that were written in English and with level 1 or 2 scientific evidence according to the SORT (Strength of Recommendation Taxonomy) criteria (6). Studies not written in English, epidemiological studies, and publications with level 3 scientific evidence according to the SORT criteria were excluded (6).

## Results

We identified 311 studies (181 after removing duplicates) from the initial search of the different databases: 169 studies in PubMed-MEDLINE, 141 in Scopus and one in the Cochrane Library. A total of 143 studies were subsequently discarded after reading the title, and the 38 remaining articles were screened by reading the abstract. Of these articles, 31 were discarded because they were related to the clinical reclassification of entities according to the new classification or did not offer relevant data in relation to the new classification.

**Figure 1:**
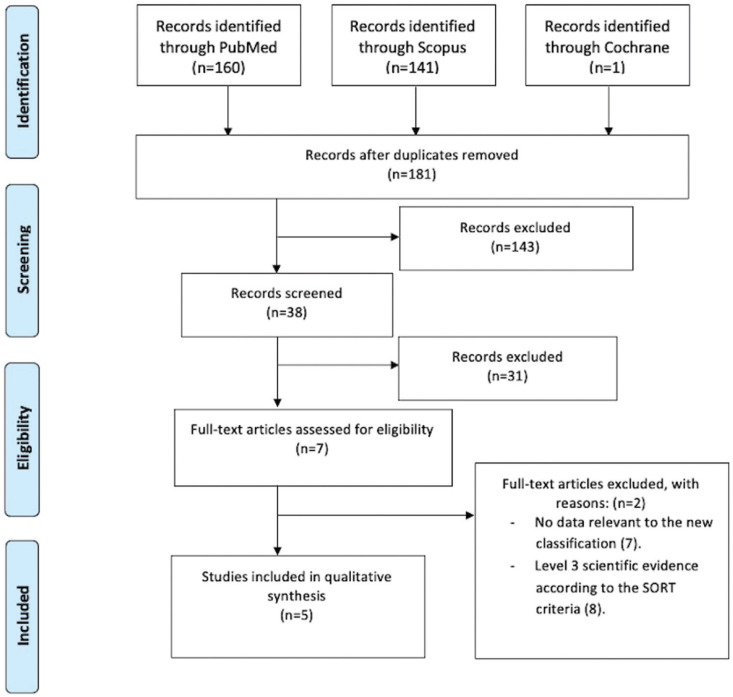
Flow-chart of the review process modified from the PRISMA statement.

Of the remaining 7 articles assessed for eligibility based on full-text reading, two were excluded because they did not contribute any relevant information relating to the new classification (7) or had level 3 scientific evidence according to the SORT criteria (8) and did not meet the inclusion criteria. Five articles were finally included in our study (4,9-12). Figure 1 shows the flowchart of the review process according to the PRISMA criteria (13).

The studies were stratified according to their level of scientific evidence using the SORT criteria (6), resulting in three articles with level 1 evidence (4,9,11) and two with level 2 evidence (10,12).

The latest classification of odontogenic cysts (5) is not complex and, as pointed out above, is similar to the previous classification of 1992 (2), used for comparison purposes. Table 1 shows both classifications (2,5), with the addition of simple bone cysts and aneurysmal bone cysts under the term pseudocysts – which in the original WHO classifications (2,5) were found in different sections within the same chapter.

**Table 1:**
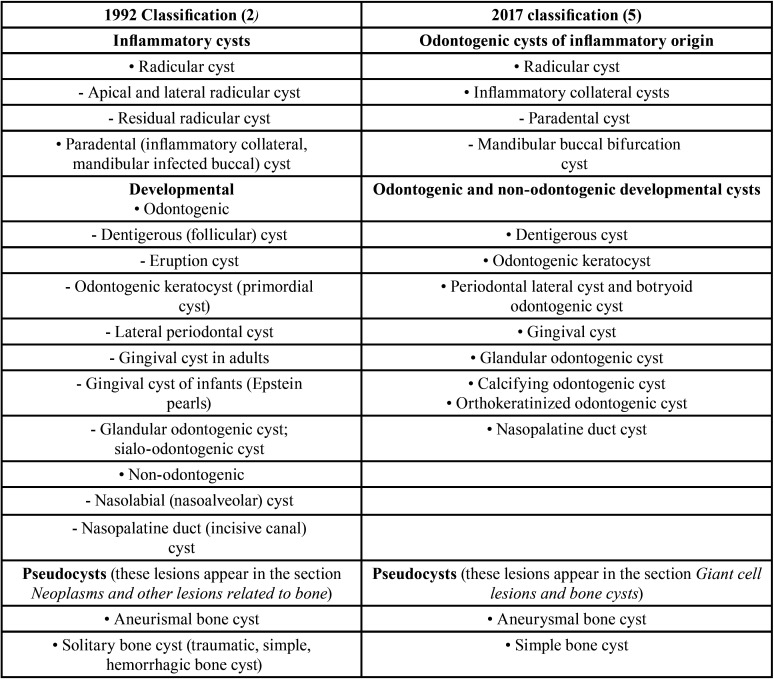
Comparison of 1992 and 2017 WHO classificationsof odontogenic and non-odontogenic cysts.

In the 2017 classification (5), in contrast to the 1992 classification (2), variants were avoided for some of these lesions such as “residual”, “apical” and “lateral” cysts, for although they are well recognized clinico-pathologically, they did not constitute a single entity and did not influence the approach to treatment (4).

The most important change in this classification (5) is the reclassification of keratocystic odontogenic tumors and calcifying cystic odontogenic tumors into odontogenic keratocysts and calcifying odontogenic cysts, respectively. Nevertheless, keratocystic odontogenic tumor is accepted as being synonymous of odontogenic keratocyst (5). Likewise, orthokeratinized odontogenic cysts are recognized as a single entity and not as a variant of odontogenic keratocysts with orthokeratosis (5).

Regarding pseudocystic lesions, aneurysmal bone cysts and simple bone cysts (also known as traumatic bone cysts, hemorrhagic bone cysts, solitary bone cysts and unicameral bone cysts) appear in the section referred to “Giant cell lesions and bone cysts” (5). In the 1992 classification these entities were included in the section of “Neoplasms and other bone lesions” (2).

The pseudocystic entity known as Stafne bone cavity has not been described in any WHO classification.

## Discussion

We analyzed the major features of the lesions that have been modified in the 2017 classification (5).

-Odontogenic keratocyst (OK)

In 1958, Philipsen (14) was the first to describe the entity known as odontogenic keratocyst, also referred to as primordial cyst. It is currently accepted that these cysts arise from remaining tissues of the dental lamina that persist in subepithelial connective tissue and bone tissue once odontogenesis has concluded (15). OKs are related to Gorlin-Goltz syndrome or nevoid basal cell carcinoma syndrome (16).

In the previous edition (3), the decision was made to classify OK as a neoplastic entity, based on certain features such as “aggressive growth”, recurrence after treatment, a high proliferation index (high Ki-67 expression) and mutations of the PTCH gene (3,9-12). These PTCH mutations were the most influential factor in this decision, because they have been documented in up to 85% of all syndromic OKs (related to Gorlin-Goltz syndrome) and in nearly 30% of all non-syndromic Oks (9,10).

Gorlin-Goltz syndrome is directly related to PTCH gene mutations, and all the cells of affected patients would carry the mutation; consequently, detecting these mutations in OK could be of predictive utility (9).

The full reasons behind this modification are not clear, though perhaps the experts intended to classify mutated OKs as neoplasms and non-mutated OKs as cystic entities (9,10). Additionally, PTCH gene mutations were documented in orthokeratinized odontogenic cysts and dentigerous cysts (17–19) Apart from PTCH gene mutations, other mutations documented in OK are: p53, CDKN2A, TP53, MCC, CADMI and FHIT (20-22).

A number of studies (23,24) have reported spontaneous regression of OK after decompression - a fact that would not be congruent with a tumor process, since the definition of neoplasm includes the concept of autonomous growth of the lesion once the causal stimulus has been eliminated (9,11).

On the other hand, there have been reports of dermal cysts histologically identical to OKs; their reclassification as neoplasms has not yet been recommended, and they are currently referred to as cysts (9).

The new classification of 2017 (5) has not ruled out the possibility that OKs may constitute tumors, though there is still not enough scientific evidence justifying their consideration as neoplasms (4,9-11).

The term primordial cyst, previously used as synonymous of some OKs, has also been discarded.

-Calcifying odontogenic cyst

Calcifying odontogenic cysts are related to ghost cell lesions (25); in 1992 (2) they were regarded as non-neoplastic cystic lesions, though the existence of a neoplastic solid variant called “dentinogenic ghost cell tumor” was suggested.

In the 2005 edition (3), a lesion called “calcifying cystic odontogenic tumor” was described as a cystic neoplasm, and “dentinogenic ghost cell tumor” was maintained as a differentiated entity for the solid forms.

In a multicenter study, Ledesma-Montes *et al*. (25) observed that up to 85% of all ghost cell lesions were simple cysts, presenting either isolatedly (65%) or associated to odontomas (20%). Some of them presented ameloblastoma-like proliferation areas, and only up to 5% of the lesions were solid and could be considered true dentinogenic ghost cell tumours. Moreover, it has been recognized that cystic lesions rarely recur and have a completely benign behavior (11).

In this latest edition (5) the decision was made to again reclassify these lesions, naming the cystic variant as “calcifying odontogenic cyst” and the tumor variant as “dentinogenic ghost cell tumor”. Calcifying odontogenic cyst is defined as a simple cyst lined by an ameloblastoma-like epithelium containing local accumulations of ghost cells (5).

-Odontogenic cysts of inflammatory origin

In this section there have been some significant changes in the classification, since the last time these lesions were grouped was in the 1992 classification (2).

In the 1992 classification (2) these lesions were classified within the group of inflammatory odontogenic cysts, which was divided into radicular cysts with their different variants and paradental cysts. In this latest classification (5) we also find radicular cysts, representing the most frequent maxillary cysts of inflammatory origin and being related to non-vital teeth, as well as a group with a new denomination - collateral inflammatory cysts - that modify the group of paradental cysts.

With regard to radicular cysts, and although not cited as an entity in their own right, residual cysts are described as those lesions that remain in the jaws after extraction of the tooth causing the radicular cyst (5). Residual cyst has been described in the literature as being one of the lesions most frequently related to odontogenic cyst malignancy, along with dentigerous cyst and OK (26–28). Its suppression as a special clinicopathological entity in the classification therefore might not have been properly justified.

The term inflammatory collateral cysts has been proposed for the first time (5) and comprises paradental cysts and mandibular buccal bifurcation cysts. While the former are usually located in distal third molar areas, mandibular buccal bifurcation cysts originate in first and second molar eruption surface zones (11,29). The etiopathogenesis of these lesions remains unclear, though it is believed that they might originate from the reduced enamel epithelium or from the sulcular epithelium (30).

-Odontogenic and non-odontogenic developmental cysts 

Orthokeratinized odontogenic cyst was first described in 1981 as a variant of odontogenic keratocyst completely or predominantly lined by an orthokeratinized stratified squamous epithelium (31).

In this latest classification (5) it has been definitely accepted that this entity should be regarded as a single lesion independent from odontogenic keratocyst. Although its clinical presentation may be similar, it is usually located in the posterior mandibular area and radiographically appears as a well-defined unilocular radiolucency (4,32). Nevertheless, it is important to point out that orthokeratinized odontogenic cysts do not exhibit aggressive growth and are not related to Gorlin-Goltz syndrome (4,9,11). They also differ histopathologically from odontogenic keratocysts because they present superficial orthokeratosis and not corrugated parakeratosis, and the basal layer does not show a prominent palisade pattern (5,9).

New diagnostic criteria have been presented for glandular odontogenic cyst (9,11). Ten different histopathological features have been described, and a minimum of 7 of them are needed to establish the definitive diagnosis (33). In some cases, these cystic lesions can exhibit morphological features similar to those of central mucoepidermoid carcinoma. It is therefore very important to establish a correct differential diagnosis, which proves very difficult with an incisional biopsy (9,11). In this regard, MAML2 gene rearrangements that have been observed in central mucoepidermoid carcinoma could be useful for differentiating it from glandular odontogenic cyst (34). It has been documented that glandular odontogenic cysts could have an aggressive behavior and a high recurrence rate, especially if treatment consists of simple enucleation of the cystic lesion (5).

Other developmental cysts such as gingival cyst, dentigerous cyst and lateral periodontal cyst have only experienced minor changes in the 2017 classification (5,10). In the case of lateral periodontal cysts, a multilocular variant has been added, referred to as “botryoid cyst” (5).

Lateral periodontal cysts appear radiographically as a well-defined, rounded radiolucent image that is often confused with an odontogenic cyst of inflammatory origin; however, in this lesion the adjacent teeth preserve their vitality - this being a key factor in establishing the differential diagnosis (35,36).

Gingival cyst of infants and gingival cyst in adults have been grouped under the general term of “gingival cysts”(5).

Eruption cyst, which constituted a single entity in the 1992 classification (2), is considered in the new classification (5) as a variant of dentigerous cyst that is located in the soft tissues around the crown of an erupting tooth (11).

Only one type of non-odontogenic developmental cyst has been included in the 2017 classification: the nasopalatine duct cyst (5). This lesion arises on the midline of the anterior maxilla and constitutes the most frequent non-odontogenic cyst (up to 1% of the total) (37). Approximately half of the cases exhibit areas of respiratory epithelium, though only up to 10% are completely lined by this epithelium (5).

-Pseudocysts

Pseudocysts are defined as pathological cavities without an epithelial lining and with clinical and radiological findings similar to those of true cysts. Within this category we find simple bone cyst, aneurysmal bone cyst and Stafne bone cavity.

Regarding simple bone cyst and aneurysmal bone cyst, both of these entities appear in the section “Giant cell lesions and bone cysts” (5).

Simple bone cyst of the jaws is an asymptomatic bone cavity without an epithelial lining that may appear empty or with a serohemorrhagic content (38,39). It is predominantly located in the mandible and is sometimes associated to florid cemento-osseous dysplasia. The clinical and radiological differential diagnosis must include the following entities: odontogenic keratocyst, ameloblastoma, odontogenic myxoma/myxofibroma, aneurysmal bone cyst, central giant cell granuloma and fibro-osseous lesions, among others (38,40).

Aneurysmal bone cyst is currently considered to be a cystic or multicystic expansive and osteolytic neoplasm composed of spaces with a hemorrhagic content, separated by fibrous connective septae (5).

The etiopathogenesis of simple bone cyst remains unclear, though the presence of an intraosseous hematoma has been suggested to be secondary to trauma, venous obstruction, local alteration in bone growth or changes in bone metabolism. In contrast, in the case of aneurysmal bone cyst, the most widely accepted theory is probable previous trauma that would cause blood accumulation within the bone tissue (38).

Both entities are relatively infrequent in the jaws, and can be found wrongly defined in the old scientific literature as true non-odontogenic cysts or other types of bone disease (38).

Stafne bone cavity in turn has never been included in WHO texts, and is a radiolucent, unilateral asymptomatic cavity located in the posterior mandible, between the mandibular angle and the third molar, below the inferior dental canal (41). Other denominations for this lesion have been: static bone cavity, Stafne bone cyst, latent bone cyst, aberrant salivary gland defect, submaxillary salivary glandular inclusion and lingual cortical mandibular bone defect, among others (42). Currently we can find four variants of this entity, though the posterior lingual variant is generally the one that receives the name of Stafne bone cavity (41). The differential diagnosis is usually straightforward, but can become complicated when the lesion is located near apical areas of mandibular teeth - this circumstance in our opinion warranting its inclusion in this study (42). The underlying etiopathogenesis remains unclear, though most authors consider that the cavity could be related to the pressure exerted by the submandibular gland upon the mandibular lingual plate (42).

## Conclusions

The incorporation of odontogenic cysts to the World Health Organization Classification of Head and Neck Tumors (2017) confirms and completes its aim to serve as an international guide for the diagnosis and management of head and neck disorders of this kind. The information referred to some of these lesions has been significantly updated thanks to improved genetic and immunochemical characterization that had not been done in either 1992 or 2005. Currently, although it has been decided that odontogenic keratocyst remains a cystic disorder, the debate over whether it is a neoplasm or a cyst continues. This do not seem to occur with calcifying odontogenic cyst, however, where the current scientific evidence has justified its reclassification as a cystic lesion, and its solid variant - dentinogenic ghost cell tumor - is contemplated within the new classification as an odontogenic neoplasm. On the other hand, residual cyst seems to have been obviated from the classification without proper justification. This consequently opens the possibility of further changes in the next edition, particularly taking into account that this lesion exhibits common features associated with odontogenic cyst malignancy. It has also been recognized that orthokeratinized odontogenic cyst constitutes an entity in its own right and not a variant of odontogenic keratocyst. An aggressive behavior of glandular odontogenic cyst has been described and microscopic differential diagnosis from central mucoepidermoid carcinoma is still debaTable to date, even with MAML2 rearrangement.
